# STAT1 Regulates Human Glutaminase 1 Promoter Activity through Multiple Binding Sites in HIV-1 Infected Macrophages

**DOI:** 10.1371/journal.pone.0076581

**Published:** 2013-09-24

**Authors:** Lixia Zhao, Yunlong Huang, Jialin Zheng

**Affiliations:** 1 Center for Translational Neurodegeneration and Regenerative Therapy, Shanghai Tenth People’s Hospital, Tongji University School of Medicine, Shanghai, China; 2 Laboratory of Neuroimmunology and Regenerative Therapy, University of Nebraska Medical Center, Omaha, Nebraska, United States of America; 3 Department of Pharmacology and Experimental Neuroscience, University of Nebraska Medical Center, Omaha, Nebraska, United States of America; 4 Department of Pathology and Microbiology, University of Nebraska Medical Center, Omaha, Nebraska, United States of America; South Texas Veterans Health Care System and University of Texas Health Science Center at San Antonio, United States of America

## Abstract

Mononuclear phagocytes (MP, macrophages and microglia), the main targets of HIV-1 infection in the brain, play a pathogenic role in HIV-associated neurocognitive disorders (HAND) through the production and release of various soluble neurotoxic factors including glutamate. We have previously reported that glutaminase (GLS), the glutamate-generating enzyme, is upregulated in HIV-1 infected MP and in the brain tissues of HIV dementia individuals, and that HIV-1 or interferon-α (IFN-α) regulates human *glutaminase 1* (*GLS1*) promoter through signal transducer and activator of transcription 1 (STAT1) phosphorylation in macrophages. However, there are multiple putative STAT1 binding sites in human *GLS1* promoter, the exact molecular mechanism of how HIV-1 or IFN-α regulates human *GLS1* promoter remains unclear. To further study the function of the putative STAT1 binding sites, we mutated the sequence of each binding site to ACTAGTCTC and found that six mutants (mut 1,3,4,5,7,8) had significantly higher promoter activity and two mutants (mut 2 and mut 6) completely lost the promoter activity compared with the wild type. To determine whether sites 2 and 6 could interfere with other inhibitory sites, particularly the nearby inhibitory sites 3 and 5, we made double mutants dmut 2/3 and dmut 5/6, and found that both the double mutants had significantly higher activity than the wild type, indicating that sites 3 and 5 are critical inhibitory elements, while sites 2 and 6 are excitatory elements. ChIP assay verified that STAT1 could bind with sites 2/3 and 5/6 within human *GLS1* promoter in IFN-α stimulated or HIV-1-infected monocyte-derived macrophages. Interestingly, we found that rat *Gls1* promoter was regulated through a similar way as human *GLS1* promoter. Together, our data identified critical elements that regulate *GLS1* promoter activity.

## Introduction

HIV-associated neurocognitive disorders (HAND), the neurological complications of HIV-1 infection, remain prevalent despite the widening use of combination antiretroviral therapy. The underlying pathophysiology of the cognitive impairment is the neuronal damage that likely stems from prolonged inflammation in the central nervous system (CNS) [1,2,3,4]. Mononuclear phagocytes (MP, macrophages and microglia), the main targets of HIV-1 infection in the brain, play a pathogenic role in HAND through the production and release of various soluble neurotoxic factors including glutamate [5,6,7]. We recently found that glutamate, a neurotransmitter that is neurotoxic in high concentrations [8,9], is significantly increased in postmortem brain tissues collected from HIV-1 serum positive patients and HIV-1 associated dementia (HAD) patients [10]. However, the potential mechanism as well as cell source of excessive glutamate in HIV-1 patients remains elusive.

Our previous data have demonstrated that mitochondrial glutaminase (GLS), the key enzyme that converts glutamine to glutamate in the CNS, is important for glutamate production in HIV-1-infected human monocyte-derived macrophages (MDM) and microglia [7,10,11]. GLS has two isoforms, Kidney-type GLS (KGA, or glutaminase 1, *GLS1*) and Liver-type GLS (LGA, or glutaminase 2, *GLS2*). GLS1 is highly expressed in the brain [12]; it is upregulated in HIV-1 infected MDM and microglia [10,13], and in postmortem brain tissues of HAD patients [10,14]. Understanding GLS regulation in HIV-1 infection may further elucidate how HIV-1 induces neurotoxicity, therefore providing a new target for therapeutic intervention.

Because GLS play vital roles in metabolism and antioxidant function, its transcription is tightly regulated [15,16,17]. GLS regulation is complex in transcriptional, translational and posttranslational levels. Human *GLS1* promoter [14], rat *Gls1* promoter [18,19], and human *GLS2* promoter [20] were previously described. We have previously characterized human *GLS1* promoter with hallmark elements of TATA box and CAAT box and several transcription factors binding sites. Some of the transcription factors, including AP-1, NF-1 and SP-1, were predicted to constitutively regulate *GLS1* activity. Interestingly, *GLS1* promoter is also regulated by STAT1 under IFN-α stimulation or HIV-1 infection [14].

HIV-1 infection induces release of type I interferons (IFN), including multiple subtypes of IFN-α and IFN-β [14,21], which initiate the downstream signal transduction cascade after binding to their receptor, interferon α receptor (IFNAR) [22]. One of the output of the cascade is the activated STAT dimers, which translocate to the nucleus and bind with the interferon stimulated response elements (ISRE) [23] or the g-activated sequence (GAS) [24] of IFN-stimulated genes. Our previous studies showed that type I IFNs regulate the STAT pathway in HIV-1-infected MDM [25], and that HIV-1 regulates *GLS1* promoter through STAT1 activation [14]. As the first group to clone and characterize the human *GLS1* promoter, we found that both IFN-α and HIV-1 infection enhanced STAT1 binding with the *GLS1* promoter and increased *GLS1* promoter activity. The increased *GLS1* promoter activity enhanced *GLS1* expression and glutamate production [14]. To further understand how STAT1 regulates human *GLS1* promoter, we mutated the sequence of each binding site and found that there are two STAT1 putative binding sites with excitatory function and six STAT1 binding sites with inhibitory function. ChIP assay confirmed the binding of STAT1 with these putative binding sites. Studies on rat *Gls1* promoter showed similar regulation by STAT1, suggesting that *GLS1* promoter regulation by multiple STAT1 binding sites are not species-specific. Because *GLS1* is important for glutamate production, understanding its transcriptional regulation may provide a new target for therapeutic intervention in HAND.

## Results

### Two putative STAT1 binding sites are essential for human GLS1 promoter activity

**Figure 1:**
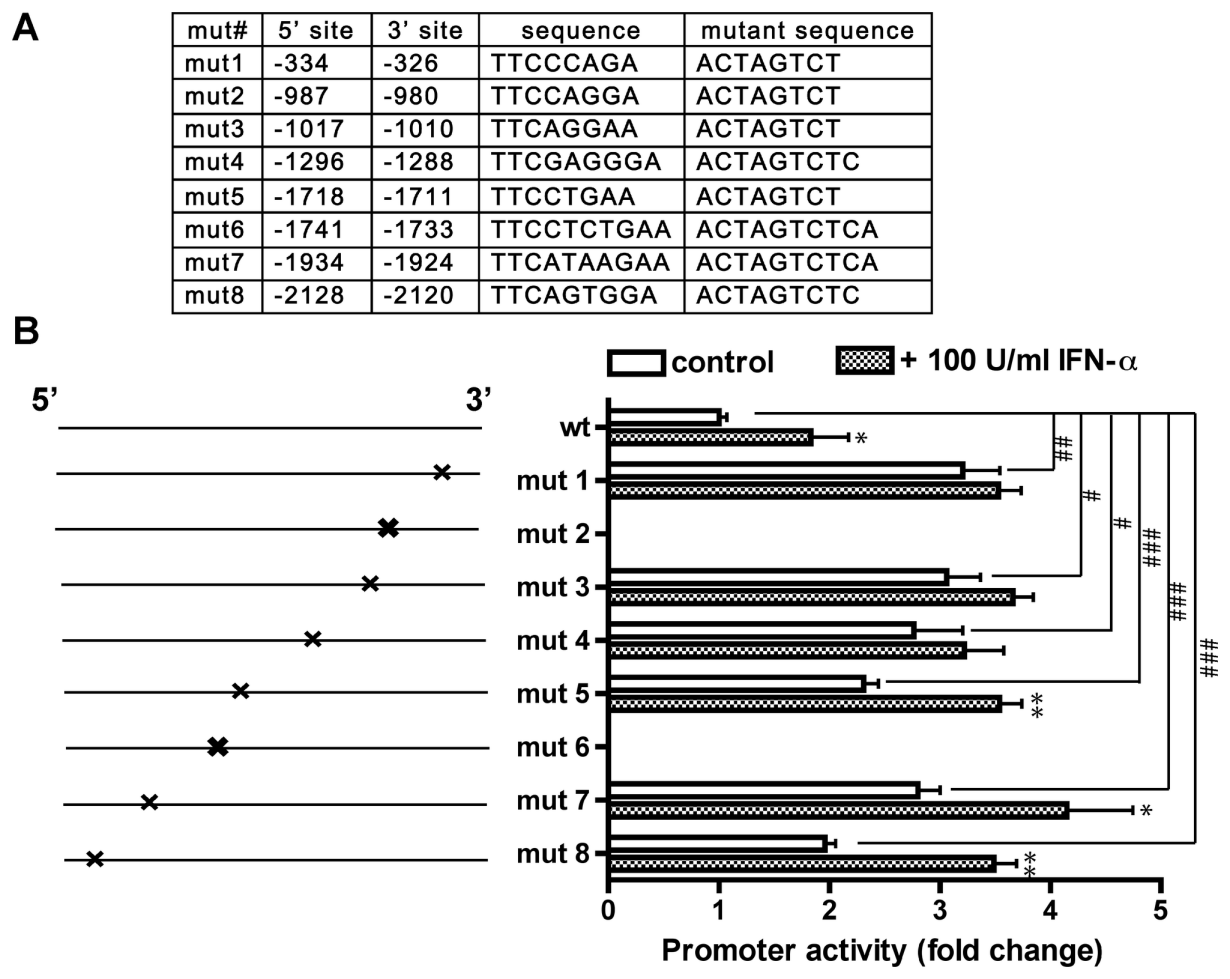
Two putative STAT1 binding sites are essential for human *GLS1* promoter activity. (A) The eight STAT1 putative binding sites and the derived mutants for these sites were listed based on their distance (base pairs) upstream from the transcription start site. (B) Schematic representation of the various promoter mutant luciferase constructs and their activities in dual-luciferase assay. HEK 293T cells were co-transfected with the human either *GLS1* promoter construct or one of the mutants, along with the Renilla luciferase construct pRL-SV40. Twenty-four hours later, the cells were treated with or without 100 U/ml IFN-α for another 24 hours. Luciferase activity in the lysates was measured by luminescence detection. Renilla luciferase was used as internal control to normalize transfection efficiency. The data are representative of three independent experiments and are the means of triplicate samples. *, p < 0.05, **, p < 0.01 when compared with the parallel control without IFN-α treatment. #, p < 0.05, # #, p < 0.01, # # #, p < 0.001 when compared with the wild type.

We have previously reported that there are eight putative STAT1 binding sites in human *GLS1* promoter [14]. To further study the mechanisms of how STAT1 regulates human *GLS1* promoter through multiple binding sites, we introduced mutants for each binding site (Figure 1A). In our previous report [14], we have demonstrated that IFN-α activates STAT1 in HEK293 cells, an effect that is similar to that of IFN-α on macrophages. These data suggest that the IFN-α-related signaling is functioning in HEK293 cells. Therefore, we performed promoter activity-luciferase reporter assay in HEK293T cells, and found that two mutants, mut 2 and mut 6, completely abolished promoter activity (Figure 1B). Furthermore, IFN-α treatment did not induce promoter activity in mut 2 and mut 6 (Figure 1B), indicating that sites 2 and 6 are essential to human *GLS1* promoter activity. In contrary to mut 2 and mut 6, all of the other mutants had 2-3 folds higher promoter activities compared with the wild type (Figure 1B). When treated with IFN-α, muts 5, 7 and 8 showed significantly increased promoter activities compared with the untreated mutants, whereas muts 1, 3 and 4 did not respond (Figure 1B). Together, these observations suggest that sites 2 and 6 are essential for *GLS1* promoter, whereas other STAT1 putative binding sites are largely inhibitory. In addition, sites 1, 2, 3, 4 and 6 seem to be important for the *GLS1* promoter in its response to IFN-α treatment.

### The inhibitory effect of the binding sites are more dominant than the excitatory sites

**Figure 2:**
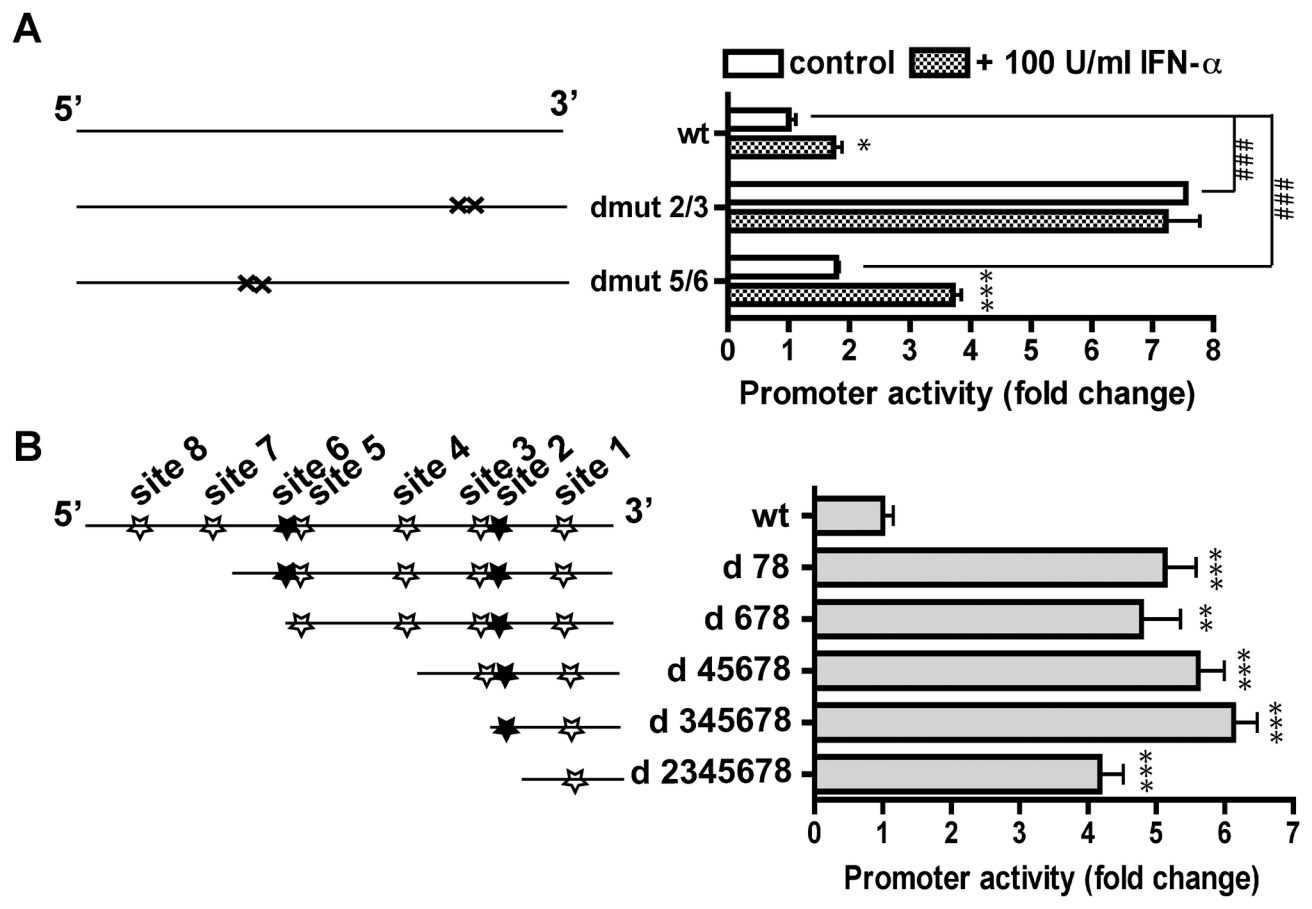
The inhibitory effect of the STAT1 binding sites to the *GLS1* promoter is more dominant than that of the excitatory sites. (A) Schematic representation of the double mutant luciferase constructs and their activities in dual-luciferase assay. After transfected with human *GLS1* promoter wild type or the double mutant constructs, cells were treated with or without 100 U/ml IFN-α for 24 hours. Firefly luciferase reporter assays were performed in HEK 293T cells. *, p < 0.05, ***, p < 0.001 when compared with the parallel control without IFN-α treatment. ###, p < 0.001 when the double mutants compared with the wild type. (B) Schematic representation of the serial deletion of the *GLS1* promoter luciferase constructs and their activities in dual-luciferase assay. Assays were performed as described in Figure 1. ***, p < 0.001 when compared with the wild type. The data are representative of three independent experiments and are the means of triplicate samples.

Given the close proximity between sites 2 and 3, sites 5 and 6, their apparent opposite effect on the basal activity of the *GLS1* promoter was quite intriguing. To determine the net effect of the excitatory site and inhibitory site in close proximity, we used a double mutant approach in the *GLS1* promoter luciferase assay in HEK293T cells. Double mutants, dmut 2/3 and dmut 5/6, had a significantly higher promoter activity compared to the wild type promoter construct (Figure 2A), suggesting that the inhibitory sites are more dominant than the active sites. Furthermore, Dmut 5/6 responded to IFN-α treatment, showing a 2-fold increase compared to the untreated mutant. In contrast, dmut 2/3 did not show any further increase of promoter activity with IFN-α treatment compared to the untreated mutant (Figure 2A), suggesting the combined effect of sites 2 and 3 are vital for the *GLS1* promoter to respond to the IFN-α treatment.

Next, we performed serial deletion of the *GLS1* promoter to determine the net effect of the loss of the STAT1 binding sites to the promoter activity. Serial deletion mutants from the 5' end, up to sites 6, 5, 3, 2, or 1 were obtained (Figure 2B). Notably, most of the promoter activities on those deletion mutants were significantly higher compared with the wild type, confirming the predominant inhibitory effect of the STAT1 binding sites (Figure 2B). Furthermore, in agreement with sites 7 and 8 mutation data in Figure 1B, deletion of sites 7 and site 8 (d 78) resulted in significantly higher promoter activity, indicating that site 7 and site 8 have a predominant inhibitory effect. The enhancing effect of d 78 on *GLS1* promoter was shared by d 678, d 45678, and d 345678. However, deletion of sites 3, 4, 5, 6, did not result in any further increase of promoter activities compared with d 78, suggesting a lack of any additive or synergistic effect by those inhibitory sites. The deletion mutants have a few implications for the functions of the individual STAT1 binding sites. For examples, deletion of sites 2-8, with only site 1 (d 2345678) left on the promoter, resulted in a lower activity compared with deletion of sites 1 and 2 (d 345678), further confirming that site 2 has an excitatory effect on the *GLS1* promoter (Figure 2B). Because IFN-α increased the wild type *GLS1* promoter activity through STAT1, the excitatory effect of site 2 may be the preferred STAT1 binding site that conveys the basal *GLS1* promoter activity as well as its response to IFN-α treatment.

### STAT1 binds directly with the GLS1 promoter in several binding sites in *IFN-α* treated and HIV-1 infected cells

**Figure 3:**
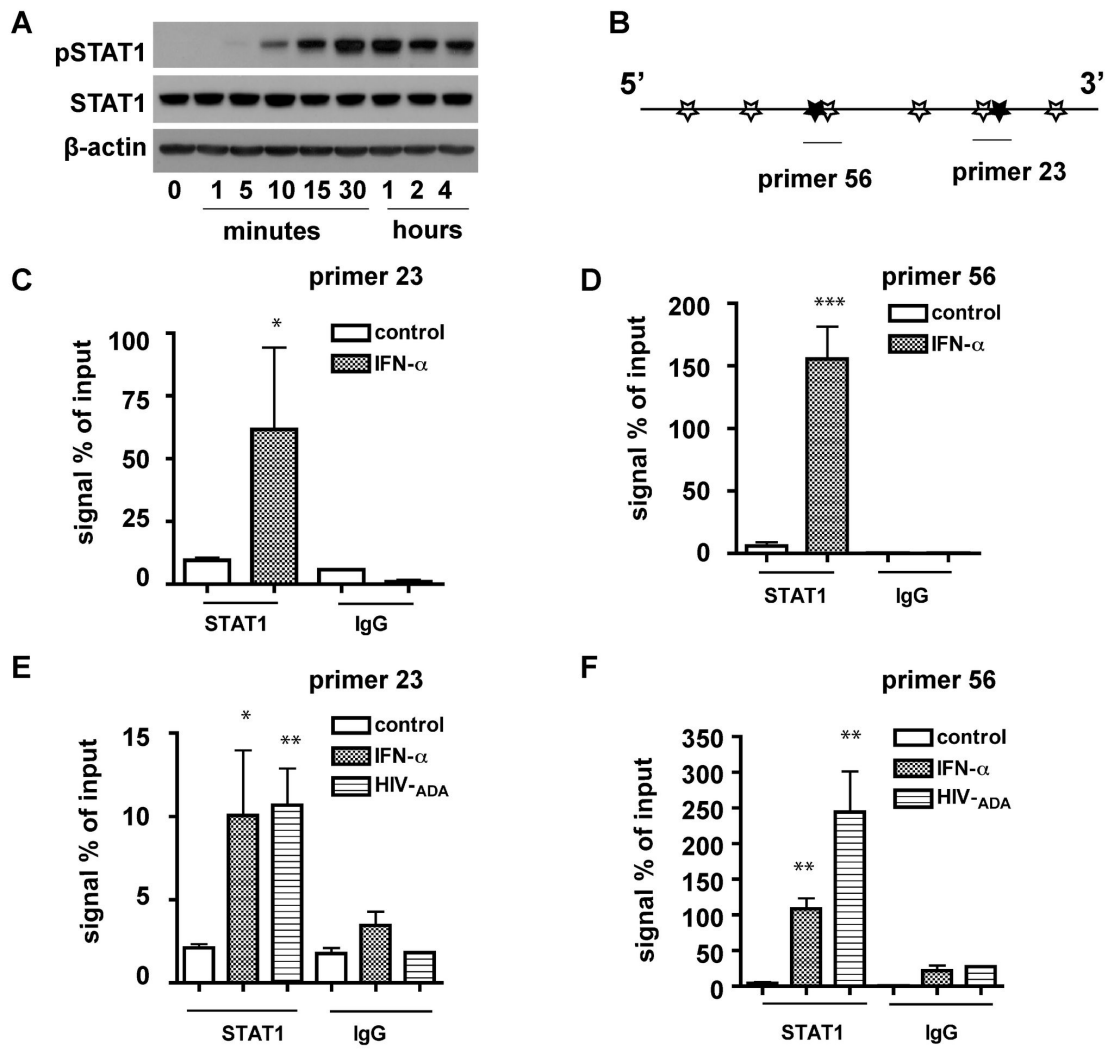
STAT1 binds directly with several binding sites of the *GLS1* promoter in IFN-α **treated and HIV-1 infected cells**. (A) THP1 cells were treated with 100 U/ml IFN-α for indicated times, then p-STAT1 (Tyr 701), and STAT1 were detected by Western blot. β-actin was used as a loading control. (B) A schematic diagram of the primers used in ChIP assay covering sites 2/3 and 5/6. (C, D) STAT1 binds with sites 2/3 and 5/6 in human *GLS1* promoter in THP1 cells. THP1 cells were treated with 100 U/ml IFN-α for one hour, then ChIP assay was performed using digested chromatin, STAT1 antibody, and IgG antibody as a negative control. Purified DNA was analyzed by real-time RT-PCR using specific primers for sites 2/3 (C) and sites 5/6 (D). The amount of immunoprecipitated DNA was normalized as a ratio to the total amount of input chromatin and shown as fold change relative to control without treatment. The data are representative of three independent experiments. (E, F) STAT1 binds with sites 2/3 and 5/6 in human *GLS1* promoter in MDM cells. MDM were treated with 100 U/ml IFN-α for one hour or infected with HIV-1_ADA_ for five days. ChIP assay was performed using STAT1 antibody as described in (C). The data are representative of three independent experiments using three different donors. *, p < 0.05, **, p < 0.01, ***, p < 0.001 when compared with the control without IFN-α treatment.

We next used ChIP assay to determine the binding of STAT1 to sites 2 and 6, which are the essential excitatory binding sites in human *GLS1* promoter. IFN-α was used to promote STAT1 phosphorylation and activation. In THP1 cells, STAT1 was phosphorylated at 10 minutes and peaked at 1 hour after IFN-α treatment (Figure 3A). Therefore, we selected 1 hour as the time point for the ChIP assay. First, we used STAT1 antibody to immunoprecipitate the protein-DNA complex in THP1 cells treated with 100 U/ml IFN-α. Subsequently, sequences of human *GLS1* promoter in the STAT1-DNA complex were semi-quantitatively determined through real time RT-PCR (Figure 3B). IgG control antibody and primers for each binding site were used to ensure the specificity of the ChIP assay (Table 1). However, due to the close proximity between sites 2 and 3 or sites 5 and 6, the primers only detected the stretches of sites 2/3 and sites 5/6 sequence but could not distinguish each of the individual site from the neighboring site. IFN-α significantly increased *GLS1* promoter sequence signal in both sites 2/3 (6-fold increase) (Figure 3C) and site 5/6 (25-fold increase) (Figure 3D) in the protein-DNA complex, suggesting that there is direct binding of STAT1 with the human *GLS1* promoter in site 2/3 and site 5/6, and the treatment of IFN-α increases the binding of STAT1 to sites 2/3 and 5/6. Since the main cellular target of type I interferons during HIV-1 infection was macrophages, we tested the effect of IFN-α on STAT1 binding with *GLS1* promoter in MDM. Similar enhancement of STAT1 binding with *GLS1* promoter was seen with IFN-α-treated MDM (Figure 3E and F). Moreover, we observed significant increase of STAT1 binding with site 2/3 and site 5/6 on *GLS1* promoter in HIV-1 infected MDM, indicating that HIV-1 infection also regulates human *GLS1* promoter through multiple STAT1 binding sites.

**Table 1:** Primers used for mutant constructs and ChIP assay.

**Name**	**Sequence**	**Purpose**
up	CGGGGTACC GGAGCAAAAAGGAAGTCGAAGAGTAGATCTGACAACCCAACCATAG	preparing wild type and all mutants
down	CCGCTCGAGGCCGCCGGGTCCGTCAGCGCCCGCTCAACAGGGGAGGATGCTCC	preparing wild type and all mutants

mutup1	CCATGAGTCTCCCCAACAGCTCGAAACTAGTCTGTGGAGGAGCCCACTGCTTCATAAATG	preparing mut1
mutdown1	CATTTATGAAGCAGTGGGCTCCTCCACAGACTAGTTTCGAGCTGTTGGGGAGACTCATGG	preparing mut1
mutup2	CAGGAAATAGTCTAAAAACATTTTTTTGACTAGTCTTGTAATGTGTATGTAGCCTCAGGG	preparing mut2
mutdown2	CCCTGAGGCTACATACACATTACAAGACTAGTCAAAAAAATGTTTTTAGACTATTTCCTG	preparing mut2
mutup3	GGATCTACTCCATTTAAACCTAATTGTACTAGTCTATAGTCTAAAAACATTTTTTTGTCC	preparing mut3
mutdown3	GGACAAAAAAATGTTTTTAGACTATAGACTAGTACAATTAGGTTTAAATGGAGTAGATCC	preparing mut3
mutup4	GCAATTTGGGAGGCCCAGGGGGTGCAGAACTAGTCTCACCAGCCTGGGCAACCTGGCGAAACCC	preparing mut4
mutdown4	GGGTTTCGCCAGGTTGCCCAGGCTGGTGAGACTAGTTCTGCACCCCCTGGGCCTCCCAAATTGC	preparing mut4
mutup5	CCTTTCCTCTGAAACTTGATGTCTCTAACTAGTCTTATATCATTACTTTGATTCATCAAC	preparing mut5
mutdown5	GTTGATGAATCAAAGTAATGATATAAGACTAGTTAGAGACATCAAGTTTCAGAGGAAAGG	preparing mut5
mutup6	GGCAGGGGTAATATTTGTTACCTACTAGTCTCACTTGATGTCTCTATTCCTGAATATATC	preparing mut6
mutdown6	GATATATTCAGGAATAGAGACATCAAGTGAGACTAGTAGGTAACAAATATTACCCCTGCC	preparing mut6
mutup7	GCCTTTAAACCTTTAAATATCTAAAACAATTACTAGTCTCAACTCAGAGAAATTAAGGGAGAAACTG	preparing mut7
mutdown7	CAGTTTCTCCCTTAATTTCTCTGAGTTGAGACTAGTAATTGTTTTAGATATTTAAAGGTTTAAAGGC	preparing mut7
mutup8	GCCTTCTCAAACAAGGGGTTAAATACTAGTCTCCTCAATTCTTCCAAATTTTGGGAG	preparing mut8
mutdown8	CTCCCAAAATTTGGAAGAATTGAGGAGACTAGTATTTAACCCCTTGTTTGAGAAGGC	preparing mut8

dmutup2/3	CTAGTCTATAGTCTAAAAACATTTTTTTGACTAGTC	preparing dmut2/3
dmutdown2/3	GACTAGTCAAAAAAATGTTTTTAGACTATAGACTAG	preparing dmut2/3
dmutup5/6	CCTACTAGTCTCACTTGATGTCTCTAACTAGTCTTATATC	preparing dmut5/6
dmutdown5/6	GATATAAGACTAGTTAGAGACATCAAGTGAGACTAGTAGG	preparing dmut5/6

d2345678	CGGGGTACCTGTAATGTGTATGTAGCCTCAGGGAATAAC	preparing deletion without sites2345678,with only site1
d345678	CGGGGTACCATAGTCTAAAAACATTTTTTTGTCCTGGAATG	preparing deletion without sites345678,with site1 and 2
d45678	CGGGGTACCCCAGCCTGGGCAACCTGGCGAAACCCCGTC	preparing deletion without sites45678,with site123
d678	CGGGGTACCACTTGATGTCTCTATTCCTGAATATATCATTAC	preparing deletion without sites678,with site12345
d78	CGGGGTACCACTCAGAGAAATTAAGGGAGAAACTGAGAGG	preparing deletion without sites78,with site123456

site2/3up	CTGTGGATCTACTCCATTTAAAC	ChIP primer for site2/3
site2/3down	GCTACATACACATTACATTCCAG	ChIP primer for site2/3
site5/6up	GTTACCTTTCCTCTGAAACTTG	ChIP primer for site5/6
site5/6down	CATGTGTAAAACATAGTCACC	ChIP primer for site5/6

rmutup1	CCGTGCGGGACACCGGGATTCCTGAAGAGCGGACGCCCACGCCCCG	preparing rGLS promoter mut1
rmutdown1	GCGTCCGCTCTTCAGGAATCCCGGTGTCCCGCACGGCGGGACGAGG	preparing rGLS promoter mut1
rmutup2	CCGCGGACTTTTTTCGGATTCCTCCTCGTCCCGCCGTGCGGGACAC	preparing rGLS promoter mut2
rmutdown2	CGGCGGGACGAGGAGGAATCCGAAAAAAGTCCGCGGTGGGGTGTG	preparing rGLS promoter mut2

### Similar regulation of STAT1 binding sites on rat Gls1 promoter

**Figure 4:**
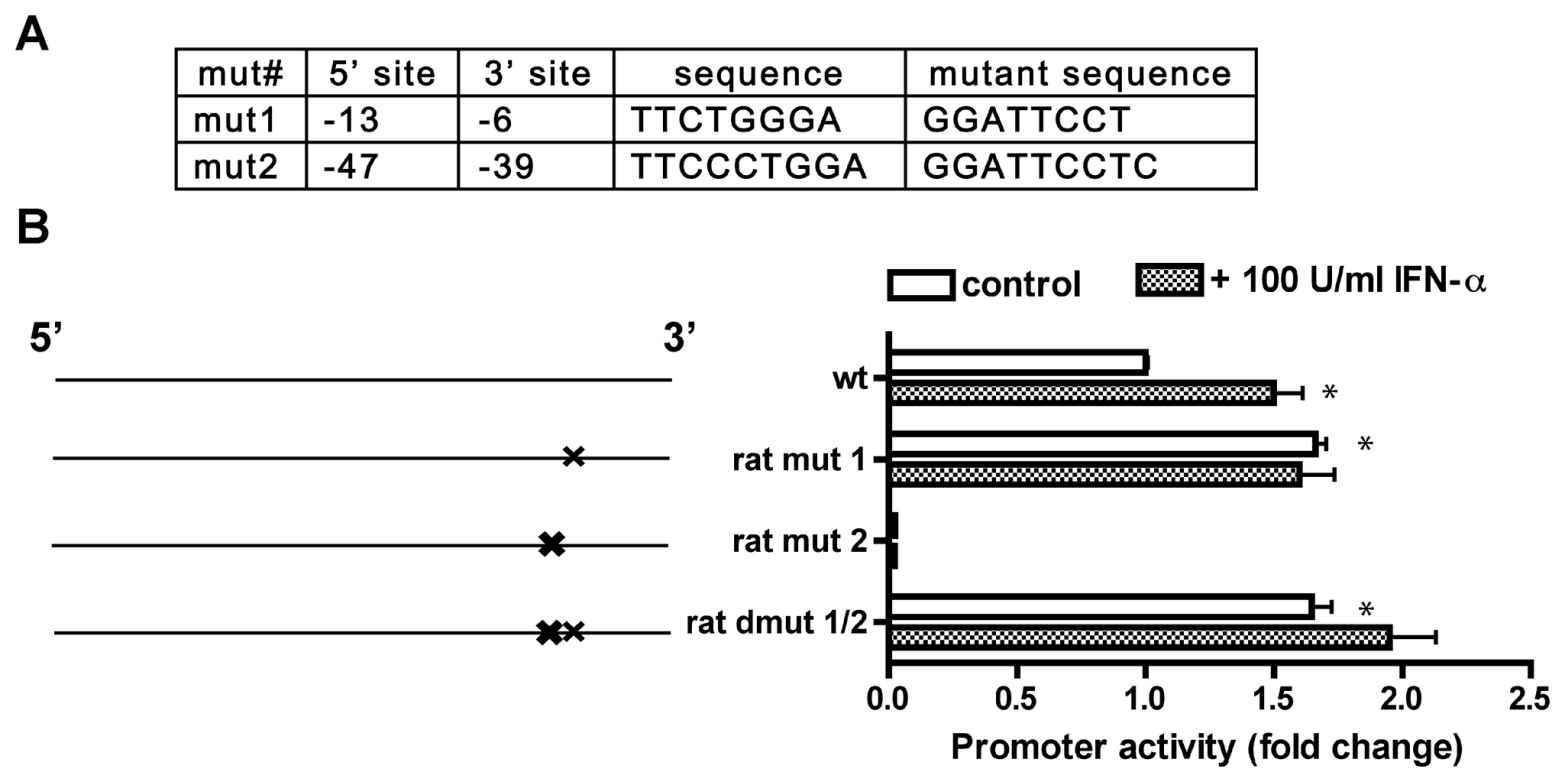
Regulation of rat GSL1 promoter activity by STAT1 binding sites. (A) Two STAT1 putative binding sites for rat *Gls1* promoter and the derived mutants for these sites were listed based on their distance (base pairs) upstream from the transcription start site. (B) Schematic representation of the various promoter mutant luciferase constructs and their activities in dual-luciferase assay. Wild type or the mutants of rat *Gls1* promoter construct-transfected cells were treated with or without 100 U/ml IFN-α for 24 hours. Luciferase promoter activity assays were performed as described in Figure 1. The data are representative of three independent experiments and are the means of triplicate samples. *, p < 0.05 when compared with the wild type.

Gene *GLS1* is highly evolutionarily conserved and the rat *Gls1* and its promoter are extensively studied [12]. Our previous study implicated six STAT1 putative binding sites in rat *Gls1* promoter, including two with close proximity -- sites 1 and 2; and ChIP assay confirmed that STAT1 directly bound to these areas [14]. To determine if these sites regulate *GLS1* promoter in a similar way with human *GLS1* promoter, we generated single site mutants rat mut 1, rat mut 2 and double mutant rat dmut 1/2 (Figure 4A). Sites 1 and 2 in rat *Gls1* promoter were chosen because their proximity closely resembles that of sites2 and 3 in human *GLS1*. Luciferase assay performed in rat astrocytes showed that rat mut 2 completely lost promoter activity compared with that of the wild type (Figure 4B), an effect similar with mut 2 and mut 6 in human cells. Furthermore, similar with mut 3 and mut 5 in human cells, rat mut 1 had a significantly higher activity compared with that of the wild type (Figure 4B). These data indicate that site 2 in rat *Gls1* promoter acts as an excitatory site, while site 1 acts as an inhibitory site. As expected, double mutant for sites 1/2 (dmut 1/2) resulted in significantly higher promoter activity (Figure 4B), indicating the inhibitory site 1 has a more dominant effect. IFN-α significantly increased promoter activity of the wild type rat *Gls1* promoter, however, both mut 2 and dmut 1/2 failed to respond to IFN-α treatment (Figure 4B), indicating that site 1/2 are vital for IFN-α response. Together, the near identical response of the *GLS1* promoter to STAT1 binding sites between rat and human suggests that the regulation of the *GLS1* promoter through STAT1 is not species-specific.

## Discussion

Little is known about how GLS expression is regulated. In our previous study [14] we demonstrated that IFN-α and HIV-1 infection specifically activated the human *GLS1* promoter through STAT1 phosphorylation and activation. The present study further elucidated *GLS1* promoter regulation by STAT1. Through single and double mutations and promoter activity assay in HEK293T cells, we found that STAT1 putative binding sites 2 and 6 were critical excitatory sites, whereas other binding sites are largely inhibitory. Furthermore, both human and rat *GLS1* promoter use similar regulation by STAT1 through multiple binding sites. Since *GLS1* converts glutamine into glutamate, which is neurotoxic when in excess levels, *GLS1* regulation through STAT1 may have an adverse effect in the CNS that are relevant to various neurological diseases.

In the human *GLS1* gene, there are TATA box and CAAT box in the first 100 bp upstream of the transcription start site (TSS) [14]. Deletion construct with only STAT1 biding site 1 left on the promoter (d 2345678) resulted in significantly higher promoter activity than the wild type promoter of human *GLS1* (Figure 2B), indicating that the deleted stretch of DNA inhibits the core promoter activity in human *GLS1* gene. The presence of eight putative STAT1 binding sites along with other constitutively active transcription factors binding sites in the *GLS1* promoter sequence make the regulation of human *GLS1* promoter extremely complex. Notably, two STAT1 binding sites, sites 2 and 6, are excitatory, whereas six other binding sites are inhibitory (Figure 1B). Note that the mutations of site 2 and 6 may change the secondary structures of promoter in addition to the disruption of the STAT1 binding. The detailed molecular mechanism of how site 2 and 6 regulate *GLS1* promoter remains to be further elucidated. We were surprised to find that both double mutants, dmut 2/3 and dmut 5/6, restored the promoter activity, indicating that the excitatory sites 2 and 6 were not as dominant as their neighboring inhibitory binding sites. It is possible that the double mutants mark secondary structure of site 2 and 6, and therefore rescue the functional loss by site 2 and 6 mutation. Importantly, the apparent opposite effects of the STAT1 binding sites on *GLS1* promoter suggest that the regulatory effect of STAT1 binding sites on the *GLS1* promoter is not uniform, and each STAT1 binding site may regulate *GLS1* promoter through differential interactions with other transcription factors [26].

Our data identified a few STAT1 binding sites that are important for the response of *GLS1* promoter to IFN-α treatment. Notably sites 1, 2, 3, 4, and 6 have inhibitory function specifically under IFN-α stimulation. Furthermore, we have checked the promoter activity after IFN-α stimulation and found that IFN-α significantly increased promoter activity in both deletion of d 78 and d 678, but not in d 345678 and d 2345678 (data not shown), indicating that site 6-8 is not necessary for the promoter to respond to IFN-α. These data are consistent with the single mutation for site 7 and 8 in their response to IFN-α (Figure 1B). Similar effect occurs for site 6 if the adjacent promoter area was modified, such as in the case of double mutants 5/6 (Figure 2A).

After phosphorylation by Janus kinase (JAK), STAT1 typically dissociates from the IFNAR and enters the nucleus [22,27]. However, reconstituting STAT1 mutant that cannot be phosphorylated at Tyr 701 in a STAT1-deficient cell line was able to mediate constitutive gene expression, suggesting unphosphorylated STAT1 or phosphorylation at sites other than Tyr 701 may enter the nucleus and bind with their DNA targets as well [28,29]. Furthermore, total STAT1 is upregulated with longer treatment of IFN-α (data not shown) or HIV-1 infection [14] and there was more precipitated promoter sequence using STAT1 antibody compared with IgG control, indicating un-phosphorylated STAT1 may bind with human *GLS1* promoter in both sites 2/3 and 5/6 (Figure 3C and D). Because pSTAT1 binds with sites 2/3 and 5/6 and the binding is increased following IFN-α treatment (Figure 3C and D) or HIV-1 infection (Figure 3E and F), the potential involvement of unphosphorylated STAT1 in the regulation of *GLS1* promoter activity may need further investigations.

Eukaryotic transcription is typically achieved through spatial and temporal control of the interactions between transcription factors and gene promoters [30]. Interestingly, it is recently reported that a conserved element, located 120 kb downstream of the *GLS1* promoter, modulates *GLS* expression through forming a chromatin loop during myogenesis [31]. This chromatin loop forms the structure basis for a regulatory element to modulate its faraway target gene transcription that otherwise cannot be reach. The distance between the STAT1 binding sites and TSS indicates that a similar loop may exist for the *GLS1* promoter. In addition, the STAT1 binding sites are conserved and often shared with other STAT family proteins, such as STAT2. Although we focused our studies on STAT1 binding sites on the *GLS1* promoter, other STAT family proteins may also bind with these sites and regulate *GLS1* promoter activity.

In conclusion, we found that STAT1 regulates human *GLS1* promoter activity through multiple binding sites. These STAT1 binding sites including two excitatory sites and six inhibitory sites, of which the inhibitory sites are more dominant. STAT1 regulation of the *GLS1* promoter and the effect of various STAT1 binding sites on promoter activity were conserved between rat and human. Because both type I IFN and STAT1 are elevated in HAD [14] and many other neurodegenerative diseases [32,33,34,35], our study may help to identify novel mechanisms as well as therapeutic interventions toward those diseases.

## Materials and Methods

### Reagents

Recombinant human IFN-α was obtained from PBL Interferon Source, Piscataway, NJ; rat IFN-α was obtained from R&D systems, Minneapolis, MN.

### Mutant and double mutant constructs

All the mutants for human *GLS1* promoter were generated using an overlap PCR method. First, two PCRs were performed using primers “mutup (1 to 8)” and “down”, or, “mutdown (1-8)” and “up”; with wild type promoter as a template. Second, PCR was performed using primer “up” and “down” with the products from the first step as templates. The final PCR product was cut by restriction enzymes KpnI and XhoI and then ligated to the pGL3-basic vector (Promega, Madison, WI). The mutants for rat *Gls1* promoter were obtained by QuikChange II site-directed mutagenesis kit (Agilent Technologies, Clara, CA). Primers used in preparing these mutants and in ChIP assay are listed in Table 1. The sequences of all the wild type and mutant constructs were confirmed by sequencing.

### Cell culture, transfection and luciferase reporter assay

HEK 293T (ATCC, Manassas, VA, USA) cells were cultured in 24-well plates in Dulbecco’s modified Eagles medium (DMEM, GIBCO Invitrogen Corp, Carlsbad, CA) with 10% heat-inactivated fetal bovine serum (FBS) (GIBCO) and an antibiotic mixture containing penicillin and streptomycin. Twenty-four hours after plating, cells were transfected with 200 ng of the pGL3-basic or *GLS1* promoter/mutant-driven Firefly luciferase reporter plasmid with Lipofectamine^TM^ LTX and PLUS reagent (Invitrogen, Carlsbad, CA). Cells were co-transfected with 5 ng of Simian Virus 40 promoter-driven Renilla luciferase (pRL-SV40) plasmid as a control for transfection efficiency. Twenty-four hours post-transfection, cells were treated with cytokines for another 24 hours; then, the Firefly and Renilla luciferase were analyzed using a Dual-Luciferase Reporter System (Promega) according to the manufacturer’s instructions. Note that IFN-α slightly decreased SV40-driven Renilla luciferase (up to 30%), a finding that is similar to an early report [36]. Since the Renilla luciferase construct was used as an internal control, caution has been made to ensure all experiments of mutants and deletions treated with IFN-α were controlled by the wild type promoter activity treated with IFN-α.

### MDM and HIV-1 infection

Human monocytes were cultured as adherent monolayers at a density of 1.1 × 10^6^ cells/well in 24-well plates and cultivated in Dulbecco’s Modified Eagles Medium (DMEM, GIBCO Invitrogen Corp) with 10% heat-inactivated pooled human serum (Cambrex Bio Science, Walkersville, MD), 50 µg/ml gentamicin, 10 µg/ml ciprofloxacin (Sigma-Aldrich) and 1000 U/ml highly purified recombinant human macrophage colony stimulating factor (MCSF, a generous gift from Wyeth Institute, Cambridge, MA). Seven days after plating, MDM were infected with laboratory HIV-1_ADA_ strain at a multiplicity of infection (MOI) of 0.1-virus/target cell. The HIV-1_ADA_ was isolated from the peripheral blood mononuclear cells (PBMCs) of an infected patient with Kaposi’s sarcoma [37]. For virus stock preparation, supernatants of HIV-1_ADA_-infected MDM were collected. The titers of the virus in the supernatants were determined as we previously described [38]. For HIV-1 infection, viral stocks were diluted into the desired MOI for overnight incubation with MDM. On the second day, medium was removed and substituted with MDM culture medium that was half-exchanged every two days. Stock virus was screened for mycoplasma and endotoxin using hybridization and *Limulus* amebocyte lysate assays, respectively. Five days after infection, HIV-1-infected and replicated uninfected MDM were harvested for ChIP and Western blot assays.

### ChIP assay

ChIP assay was performed using a SimpleChIP® Enzymatic Chromatin IP Kit (#9003, Cell Signaling Technologies) according to the manufacturer’s instructions. Quantitative PCR was performed using two pairs of primers corresponding to the STAT1 binding sites located at site 2/3 and site 5/6 in the human *GLS1* promoter. Primers used in ChIP assay are listed in Table 1. Quantifications were normalized to input.

### Statistical test

Data was analyzed as means ± standard deviation unless otherwise specified. The data were evaluated statistically by unpaired student’s t-test. Significance was considered to be less than 0.05. All assays were performed at least three times with triplicate for each.

### Ethics statement

Primary rat astrocytes were made from embryonic day 14-15 rat embryos in strict accordance with ethical guidelines for care and use of laboratory animals set forth by the National Institutes of Health (NIH). The protocol was approved by Institutional Animal Care and Use Committee (IACUC) of the University of Nebraska Medical Center (approved #: 04-097-01); MDM were used in full compliance with NIH ethical guidelines. The protocol was approved by the Institutional Review Board (IRB) of the University of Nebraska Medical Center (approved #: 162-93-FB). We have the informed written consent from all participants involved in this study.
